# Transcriptomic analysis reveals the molecular basis of photoperiod-regulated sex differentiation in tropical pumpkins (*Cucurbita moschata* Duch.)

**DOI:** 10.1186/s12870-024-04777-3

**Published:** 2024-02-06

**Authors:** Shudan Xue, Hexun Huang, Yingchao Xu, Ling Liu, Qitao Meng, Jitong Zhu, Meijiang Zhou, Hu Du, Chunpeng Yao, Qingmin Jin, Chengrong Nie, Yujuan Zhong

**Affiliations:** 1 Guangdong Key Laboratory for New Technology Research of Vegetables, Vegetable Research Institute, Guangdong Academy of Agricultural Sciences, Guangzhou, 510640 P. R. China; 2 Department of Horticulture, College of Food Science and Engineering, Foshan University, Foshan, 528000 P. R. China

**Keywords:** Photoperiod, *Cucurbita moschata*, Sex differentiation, Photoperiod-mediated flowering processes, Gibberellin signaling pathway, Ethylene biosynthetic and ethylene response pathways

## Abstract

**Background:**

Photoperiod, or the length of the day, has a significant impact on the flowering and sex differentiation of photoperiod-sensitive crops. The “miben” pumpkin (the main type of *Cucurbita moschata* Duch.) is well-known for its high yield and strong disease resistance. However, its cultivation has been limited due to its sensitivity to photoperiod. This sensitivity imposes challenges on its widespread cultivation and may result in suboptimal yields in regions with specific daylength conditions. As a consequence, efforts are being made to explore potential strategies or breeding techniques to enhance its adaptability to a broader range of photoperiods, thus unlocking its full cultivation potential and further promoting its valuable traits in agriculture.

**Results:**

This study aimed to identify photoperiod-insensitive germplasm exhibiting no difference in sex differentiation under different day-length conditions. The investigation involved a phenotypic analysis of photoperiod-sensitive (PPS) and photoperiod-insensitive (PPIS) pumpkin materials exposed to different day lengths, including long days (LDs) and short days (SDs). The results revealed that female flower differentiation was significantly inhibited in PPS_LD, while no differences were observed in the other three groups (PPS_SD, PPIS_LD, and PPIS_SD). Transcriptome analysis was carried out for these four groups to explore the main-effect genes of sex differentiation responsive to photoperiod. The main-effect gene subclusters were identified based on the principal component and hierarchical cluster analyses. Further, functional annotations and enrichment analysis revealed significant upregulation of photoreceptors (*CmCRY1*, *F-box/kelch-repeat protein*), circadian rhythm-related genes (*CmGI*, *CmPRR9*, etc.), and CONSTANS (CO) in PPS_LD. Conversely, a significant downregulation was observed in most Nuclear Factor Y (NF-Y) transcription factors. Regarding the gibberellic acid (GA) signal transduction pathway, positive regulators of GA signaling (*CmSCL3*, *CmSCL13*, and so forth) displayed higher expression levels, while the negative regulators of GA signaling, *CmGAI*, exhibited lower expression levels in PPS_LD. Notably, this effect was not observed in the synthetic pathway genes. Furthermore, genes associated with ethylene synthesis and signal transduction (*CmACO3*, *CmACO1*, *CmERF118*, *CmERF118-like1,2*, *CmWIN1-like*, and *CmRAP2-7-like*) showed significant downregulation.

**Conclusions:**

This study offered a crucial theoretical and genetic basis for understanding how photoperiod influences the mechanism of female flower differentiation in pumpkins.

**Supplementary Information:**

The online version contains supplementary material available at 10.1186/s12870-024-04777-3.

## Background

The Cucurbitaceae family predominantly exhibits monoecy, characterized by unisexual flowers, and the yield of cucurbit crops is intrinsically linked to the abundance of female flowers. Ontogenesis of female and male floral buds from a bisexual floral meristem depends on a few sex-determination mechanisms. These mechanisms can be influenced by environmental factors such as temperature, photoperiod, and nutrition, as well as the application of plant growth regulators [1–8]. Photoperiod is crucial in sex differentiation in numerous cucurbit crops [4, 9–12]. Generally, short-day (SD) conditions increase the propensity for femaleness, while long-day (LD) conditions encourage maleness [13, 14]. Despite these findings, the photoperiodic response mechanism governing sex differentiation in monoecious cucurbit plants remains elusive [15].

In general, promoting female sex differentiation in cucurbit crops is influenced by low temperatures and short photoperiods, which can impact the levels of endogenous hormones, including ethylene, auxin, and gibberellins (GA). These hormones, in turn, are crucial in shaping sex differentiation patterns [16, 17]. In recent studies, molecular mechanisms of photoperiodic flowering have been identified in various plant species such as *Arabidopsis*, soybean, and rice [18–20]. A key regulator in this process is CONSTANS (CO), regulating the expression of *FLOWERING LOCUS T* (*FT*) transcripts and integrating diverse external and internal signals into the photoperiodic flowering pathway [21]. The GA biosynthesis, perception, and transduction pathways have been found to be closely associated with sex differentiation in pumpkins [22]. Additionally, the GA-mediated signaling pathway, facilitated by the GID1-GA-DELLA complex, has also been implicated in governing photoperiodic flowering [23, 24]. DELLA proteins, which are pivotal constituents within the GA signaling pathway, physically interact with CONSTANS, a crucial flowering activator in the photoperiod signaling pathway, to modulate flowering under LD conditions in *Arabidopsis* [25].

In addition, SD conditions have been documented to enhance ethylene production by expediting the expression of *CsACS2* (1-aminocyclopropane-1-carboxylate synthase) and then increasing the occurrence of female flowers in cucumber [3, 26]. Ethylene, a prominent hormone that acts as a regulator of sex differentiation, is also a principal mediator of responses to diverse environmental signals. A correlation between photoperiod and ethylene emission in flower sex differentiation exists, with detectable ethylene peaks occurring during the middle of the light period under SD conditions surpassing those under LD conditions [3]. Furthermore, Ikram et al. [17] confirmed that the expression of *CsACS2*, *CsETR1* (*ethylene response* 1), and *CsCaN* (calcium-dependent nuclease) genes associated with ethylene production was affected by photoperiod and further influenced female flower determination. However, the exact mechanisms through which photoperiod affects sex differentiation remain unclear.

Pumpkin (*Cucurbita moschata* Duch) is an essential tropical Cucurbitaceous vegetable crop. It is sensitive to photoperiod, which restricts its cultivation period in regions like South China, where it can be grown from February to April due to its photoperiod sensitivity. However, in North China’s temperate zones, it is rarely cultivated because of this photoperiod sensitivity. Overcoming this photoperiod sensitivity would enable *C. moschata* to adapt to higher latitudes and expand its geographical cultivation range. Prior investigations involved high-density linkage mapping, leading to the identification of significant quantitative trait locus (QTL) related to early flowering and photoperiodic flower traits in *C. moschata* [27, 28]. Additionally, studies on the Cucurbitaceae family have explored the impact of photoperiod on sex differentiation, focusing on the Xishuangbanna cucumber, which is strictly an SD plant. Recent genetic and transcriptomic analyses have revealed the molecular underpinnings of photoperiod-regulated flowering in Xishuangbanna cucumber. These studies have identified a significant QTL called DFF1.1, which is associated with the candidate gene *CsaNFYA1*, and is responsible for regulating the days to first flowering in XIS cucumbers [29–32]. While there are certain reports on photoperiod-sensitive Xishuangbanna cucumbers and pumpkins within the Cucurbitaceae family, the depth of research is relatively limited, and the core genes and mechanisms remain unclear.

This study focused on a specific type of pumpkin “miben”, which is highly inbred and insensitive to photoperiod (PPIS, photoperiod-insensitive). This line can flower and produce fruits even under LDs [33]. In contrast, the ordinary “miben” line, highly inbred (PPS, photoperiod-sensitive), contains a limited number of female flowers and is unable to yield fruit under LDs. Both of these germplasms normally produce female flowers under SD conditions. This study aimed to identify genes responsible for the adaptation of *C. moschata* to high latitudes. The differences in photoperiod sensitivity between two distinct germplasms, PPS and PPIS, were examined to achieve this. The study contributed to the understanding of how the photoperiod influences sex differentiation in the Cucurbitaceae family.

## Materials and methods

### Plant materials, growth conditions, and tissue collection

PPIS and PPS pumpkins are highly inbred lines of *Cucurbita moschata* Duch. To identify whether photoperiod has a direct impact on the sex differentiation, the two lines were grown in two greenhouses with different photoperiod treatments: SD (9 h/15 h, day/night) and LD (15 h/9 h, day/night) at the same temperature of 24 °C with a light intensity of 5500 lx. Shoot apices, consisting of the bud, one connected true leaf, and stem, were collected from six individual PPIS or PPS plants for each photoperiodic treatment. These samples were collected after about 3 weeks of sowing and were harvested at 09:00 am. They were rapidly frozen in liquid nitrogen and stored at − 80 °C for subsequent RNA-seq analysis and GA detection.

### RNA isolation, sequencing, and bioinformatic analyses

Total RNA was extracted from the frozen samples using TRIzol (Invitrogen, Canada) following to the manufacturer’s protocols. The quality and integrity of the 12 RNA samples were assessed using an Agilent Technologies 2100 Bioanalyzer (Agilent Technologies, Palo Alto, CA) and a Nanodrop NanoPhotometer (Nanodrop Technologies, Wilmington, Del). RNA libraries were constructed for all samples and sequenced using the Illumina HiSeq2500 platform. The high-quality reads were mapped to *C. moschata* genome after removing adapter sequences and trimming low-quality reads using SOAPnuke v1.5.2 (http://www.cucurbitgenomics.org/) and the HISAT2 software [34]. Reads were assembled and merged using StringTie software [35]. New sequences and mapped reads were subjected to a blastx alignment against protein databases, including Swiss-Prot, Gene Ontology (GO), Kyoto Encyclopedia of Genes and Genomes (KEGG), Non-Redundant (NR), Clusters of Orthologous Groups (COG), and euKaryotic Orthologous Groups (KOG), to determine the most significant sequence similarities, facilitating protein functional annotation and classification. The fragments per kilobase of transcript per million mapped reads (FPKM) value serves as a measure to signify the expression abundance of the respective genes.

### Differentially expressed genes and functional enrichment analyses

Following the calculation of transcript levels for each gene, a differential expression analysis was performed using edgeR [36] to identify the differentially expressed genes (DEGs). The false discovery rate (FDR) was used to determine the threshold of the *P* value in multiple tests and analyses. A threshold of FDR ≤ 0.01 and an absolute value of |log_2_(fold change)| ≥1 were used to evaluate the significance of the gene expression differences. GO enrichment analysis for DEGs was conducted using the R package topGO [37].

### GA measurement

The samples of shoot apices from PPIS and PPS germplasms growing under LD and SD conditions were ground to a fine powder. About 2 g of this powder was used for hormone measurement. The quantification of endogenous bioactive GAs followed the method described by Chen et al. [38], which involved a derivatization approach coupled with nano-LC-ESI-Q-TOF-MS analysis.

### RT-qPCR analysis

Ten genes showing significant differences associated with sex differentiation in RNA-seq-based expression profiles among PPS_LD, PPS_LD, PPIS_SD, and PPIS_LD were subjected to qRT-PCR analysis. cDNA synthesis was conducted using the identical RNA samples employed in the RNA-seq analysis. The synthesis was performed using the TUREscript cDNA Synthesize Kit (Aidlab, China) following the manufacturer’s protocol. Subsequently, RT-qPCR was performed in 96-well plates using a CFX Connect Real-Time PCR Detection System (Bio-Rad, USA) and the 2×SYBR Green qPCR Mix Kit (Aidlab, China). Three biological replicates and three technical replicates were conducted in the experiments. Relative quantitative analysis of the data was performed using the 2^−△△Ct^ method, with β-actin serving as an internal control gene. The precise RT-qPCR primers for 10 genes related to photoperiod-mediated flowering processes, GA signaling pathway, and ethylene biosynthetic and ethylene response pathways are listed in Table S1.

## Results

### Identification of the photoperiod insensitivity of PPIS pumpkin germplasm

**Fig. 1:**
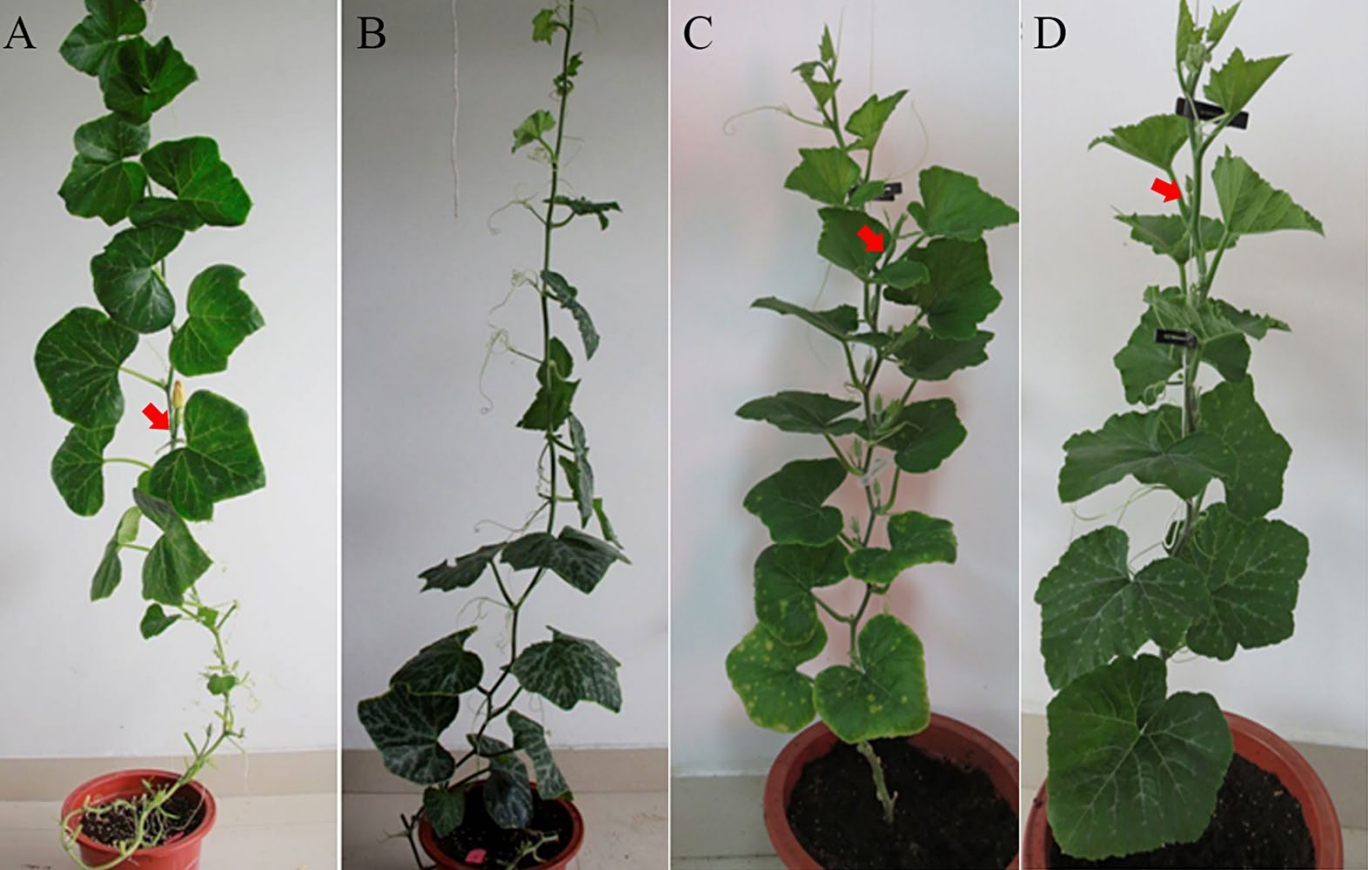
Flowering difference in PPIS versus PPS plants growing under moderate LD and SD conditions: (**A**) PPIS_LD, (**B**) PPS_LD, (**C**) PPIS_SD, (**D**) PPS_SD. Red arrows indicate first female flower bud

PPIS (photoperiod-insensitive) and PPS (photoperiod-sensitive) lines were grown in a greenhouse at 24 °C under distinct day-length conditions, 9 h of light followed by 15 h of darkness (L:D) and 15 h of light followed by 9 h of darkness (L:D), to compare the sex differentiation response to photoperiod. The two germplasms PPIS and PPS, showed different flowering phenotypes under distinguishing daylight conditions. Under SD conditions, both PPIS and PPS germplasms displayed almost identical numbers of female flowers and exhibited a similar node for the first female flower. However, under LD conditions, PPIS exhibited similar results, whereas PPS did not produce any female flowers. This suggests a more stringent photoperiod requirement for inducing female flowers in PPS (Fig. 1; Table 1).

**Table 1:** Comparison of number of female flowers and node of first female flower under SD and LD conditions

	SD 9 h (D)/15 h (N), 24˚С		LD 15 h (D)/9 h (N), 24˚С	
	**Node of first female flower**	**Total number of female flower**	**Node of first female flower**	**Total number of female flower**
PPIS	20 ± 1	4 ± 1	21 ± 1	4 ± 1
PPS	21 ± 1	4 ± 1	> 50	0 ± 0

The data were the average of 10 repeats ± SD. The female flowers within 50 nodes were counted. D, day; N, night

### Generation of transcriptome data between PPIS and PPS pumpkins under different photoperiods

The transcriptome data of four samples (PPS_ SD, PPS_LD, PPIS_SD, and PPIS_LD) were compared to gain insights into the alteration of sex differentiation due to changes in photoperiods of PPIS and PPS pumpkins. The RNA-seq of the 12 libraries produced 96.18 Gbp clean data, with each library containing more than 7.5 Gbp clean data. The Q30 scores ranged from 92.68 to 94.88% in the clean reads of the 12 libraries. The libraries of the PPS and PPIS samples under SD and LD conditions produced about 47,759,066 to 59,059,336 of total raw reads, of which from 94.04 to 95.47% of reads in 12 libraries could be mapped to the *C. moschata* genome (http://www.cucurbitgenomics.org/) (Table S2). In addition, 707 novel genes were detected, of which 487 genes were functionally annotated. The novel genes were annotated by sequence alignment with the public protein databases, including Swiss-Prot, GO, KEGG, NR, COG, and KOG.

**Fig. 2:**
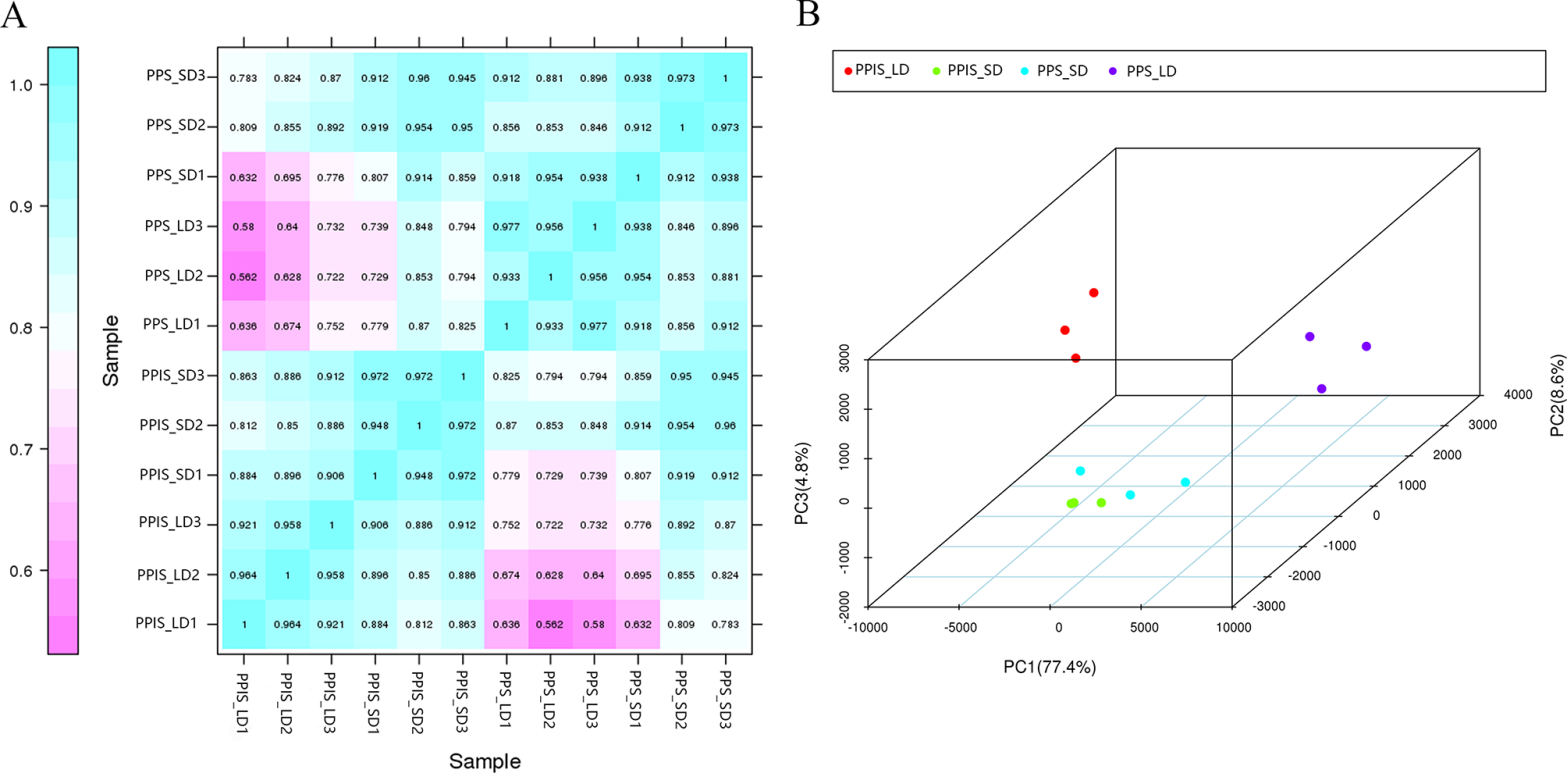
Pearson correlation analysis and principal component analysis were conducted on 12 samples. (**A**) Pearson correlation illustrating the gene expression relationships among the 12 samples. (**B**) Principal component analysis plot displaying the clustering of RNA sequencing data for all sample types

After removing the low-quality reads, the total number of clean reads for the 12 libraries was 23,879,533 to 29,529,668 (Table S2). The gene expression correlation analysis was conducted among the 12 samples to assess the relationships between samples and the validity of the sample collection. The Pearson’s correlation coefficients between replicate samples were > 0.9 and the PCA showed that the three biological replicates of one group were clustered together. Furthermore, the PCA revealed that the samples from PPIS_LD, PPIS_SD, and PPS_SD formed a single cluster, while the PPS_LD samples constituted a distinct cluster. Notably, the PPS_LD samples had a unique profile, as they lacked female flowers, setting them apart from others (Fig. 2).

### GO enrichment and metabolic pathway analysis

**Fig. 3:**
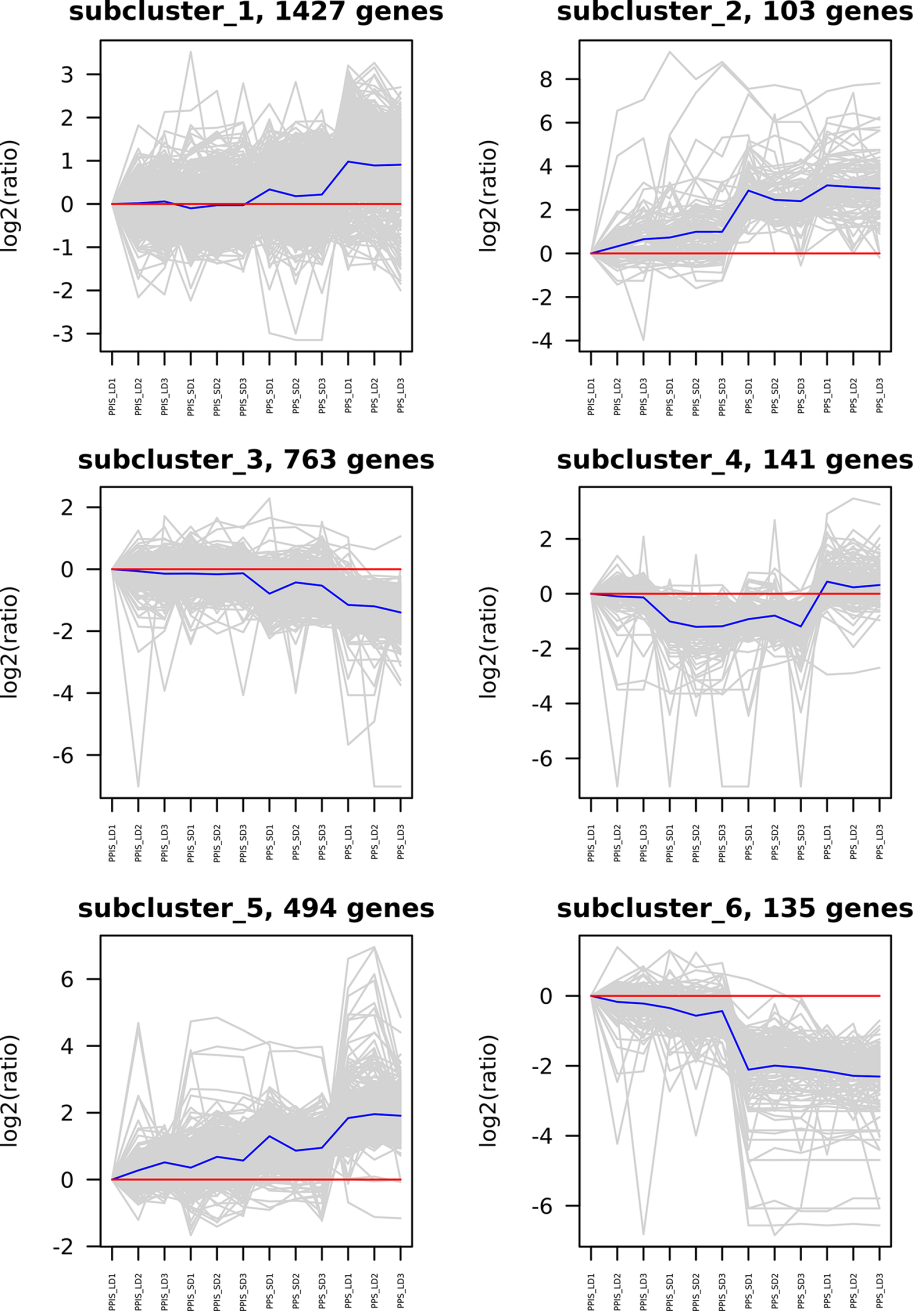
Gene expression patterns were obtained by hierarchical clustering analysis. Differentially expressed genes (DEGs) among four groups were categorized into six clusters depending on their expressions. Levels of gene expression are represented along the *y*-axis as log2(ratio), and four groups were represented along the *x*-axis as PPIS_LD, PPIS_SD, PPS_SD, and PPS_LD

**Fig. 4:**
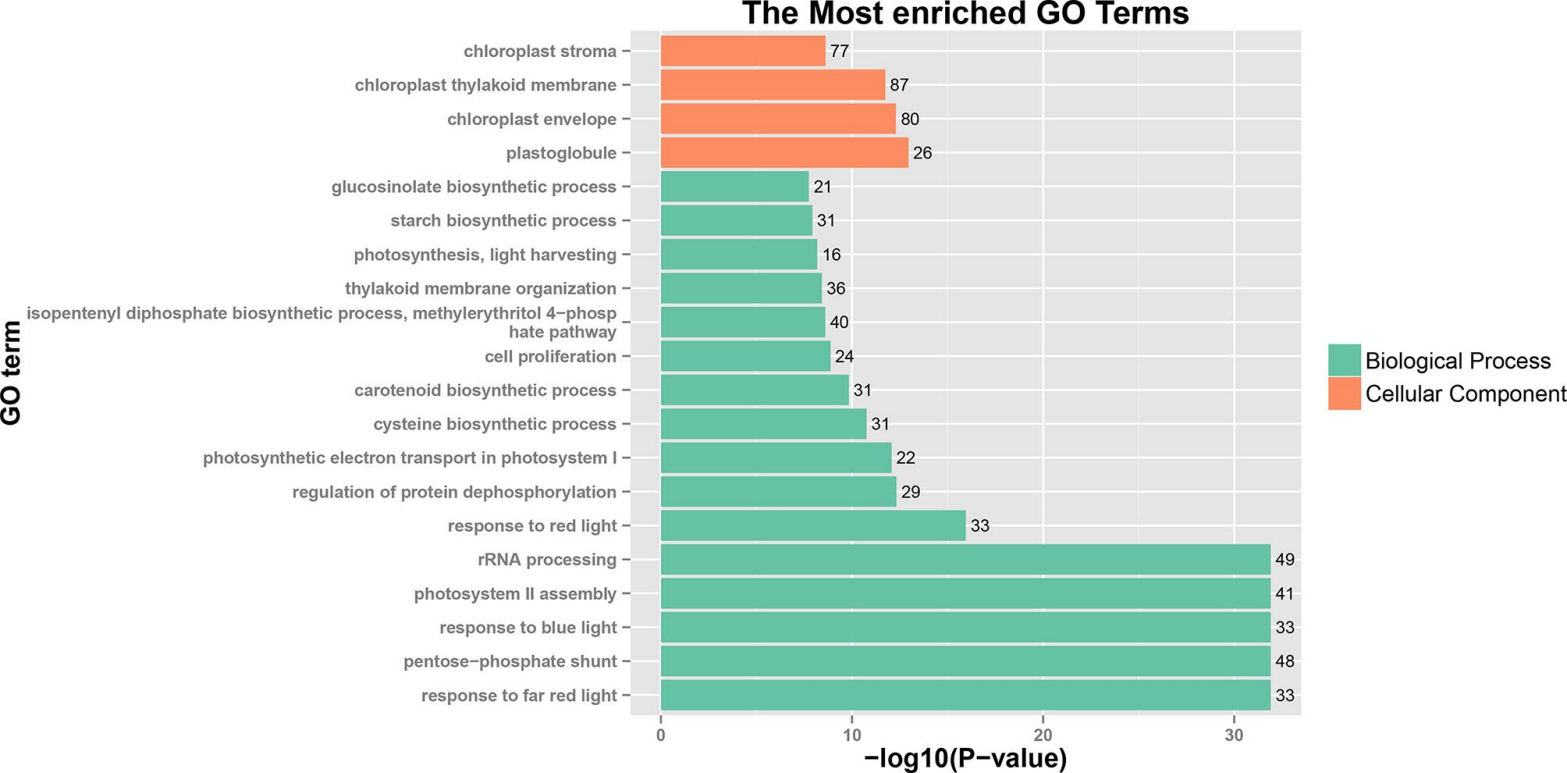
Gene ontology enrichment analysis of the genes of subclusters (1, 3, and 5) based on the biological process, molecular function, and cellular component

A cutoff parameter of FDR < 0.01 and |log_2_(Fold change)| ≥1 was used, and 156 DEGs (68 upregulated and 88 downregulated) in PPIS_LD versus PPIS_SD, 2574 DEGs (1808 upregulated and 766 downregulated) in PPIS_LD versus PPS_LD, 449 DEGs (253 upregulated and 196 downregulated) in PPIS_SD versus PPS_SD, and 1071 DEGs (837 upregulated and 234 downregulated) in PPS_LD versus PPS_SD were identified employing DESeq version 2 (Table S3). The DEGs in the PPS line under different photoperiod lengths were the main effector genes that responded to photoperiod-regulated sex differentiation. However, these genes did not respond to photoperiod changes in the PPIS line. As the sex differentiation phenotype of the PPS_LD group significantly differed from the other three groups, hierarchical clustering analysis was performed based on the DEGs among the four groups (Fig. 3). The results revealed six distinct subclusters of DEGs, and their expression trends are demonstrated in Fig. 1D. The expression trend of cluster 1 (1427 DEGs) and cluster 5 (494 DEGs) was similar, with PPS_LD significantly higher than the other groups, while the other groups had little difference. Cluster 3 (763 DEGs) showed the opposite expression trend, with PPS_LD significantly lower than the other three groups (Table S4). GO enrichment analysis of subclusters 1, 3, and 5 were performed to reveal the major functional categories represented in the genes involved in photoperiodic sex differentiation, which revealed that the DEGs were predominantly enriched in photosynthesis, light harvesting, photosynthetic electron transport in photosystem I, response to red/blue light, response to far-red light, and photosystem II assembly (Fig. 4).

### DEGs associated with photoperiodic flowering pathway

**Fig. 5:**
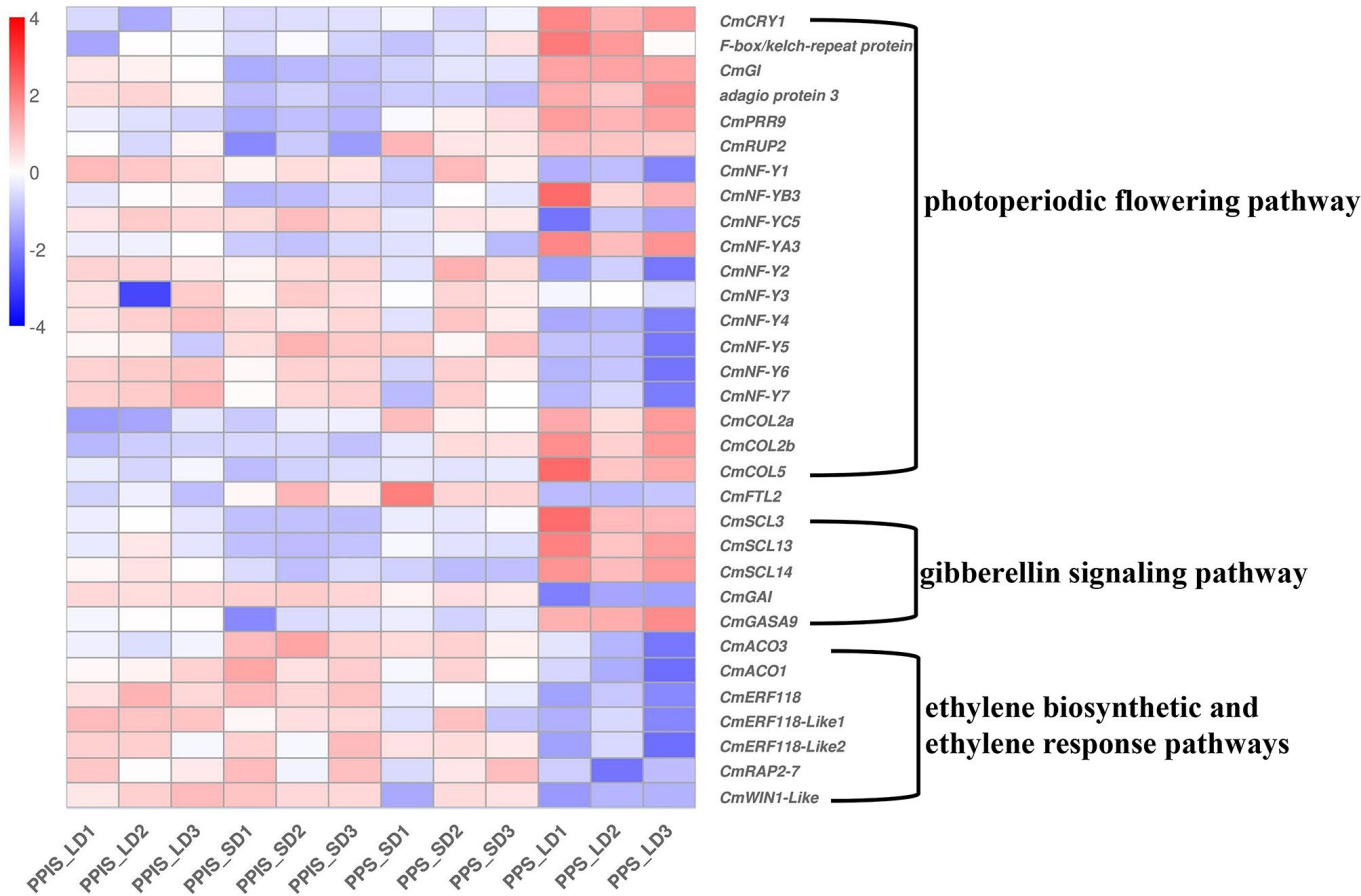
DEGs from subclusters (1, 3, and 5) are associated with the photoperiodic flowering pathway, GA signaling pathway, and ethylene biosynthetic and ethylene response pathways

The mechanisms behind photoperiodic flowering can be divided into light input, circadian clock, and output. Photoperiodic information can affect the expression of photoreceptors, NUCLEAR FACTOR Y (NF-Y) TFs (which act as positive regulators of photomorphogenesis), and CONSTANS (CO). More DEGs related to the photoperiodic flowering network were detected. Notably, two classes of blue-light photoreceptors, cryptochrome (*CmCRY1*) and F-box/kelch-repeat protein (*CmCh12G000540*), as well as genes associated with the circadian rhythm/clock, such as *CmGI* (protein GIGANTEA-like), adagio protein 3 (*CmoCh04G011030*), pseudo-response regulator 9 (*CmPRR9:CmoCh14G022280*), and E3 ubiquitin–protein ligase COP1-like (*RUP2*), all showed a higher expression level in PPS_LD. NF-Y TFs involved in light perception controlled the photoperiod-dependent flowering by interacting with CONSTANS (CO) [39–41]. The expression profile showed that most NF-Y family genes were downregulated in PPS_LD (without female flower), consistent with previous studies showing that NF-Y had a positive impact on the flowering transition process [29, 42]. CO serves as a central hub in integrating diverse external and internal signals into the photoperiodic flowering pathway [21]. For SD plants, CO was suggested to promote flowering under inductive SD conditions while delaying it under LD conditions [43]. Three CO homologous DEGs were observed, with *CmCOL2a* and *CmCOL2b* belonging to cluster 5 and *CmCOL5* belonging to cluster 1, showing significant upregulation in PPS_LD compared with the other three groups. However, *FLOWERING LOCUS T* (*CmFTL2*) was barely expressed in PPS_LD, which did not produce female flowers (Fig. 5, Table S5).

### DEGs mainly enriched in the gibberellin signaling pathway rather than the synthetic pathway

**Fig. 6:**
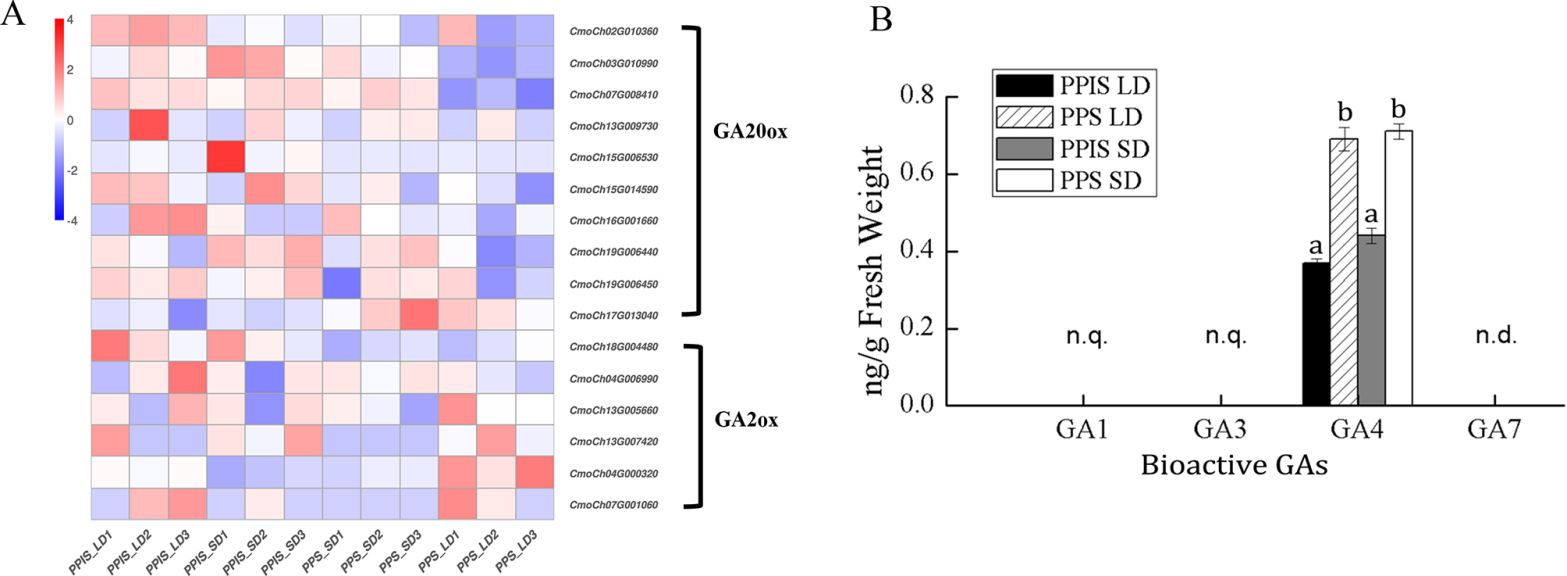
Comparative analysis of the expression of *GA20ox* as gibberellin (GA) biosynthetic genes and *GA2ox* as the GA-deactivating enzyme, and the content of four kinds of GA in PPS and PPIS seedlings under LD and SD treatments. The GA levels are normalized to ng^.^g^–1^ F.W. n.d., not detected; n.q., not quantified. In all cases, the data are represented as means ± SD (*n* = 3). Values followed by the same letter were not significantly different (*P* > 0.05). This determination was made by a one-way analysis of variance followed by a post hoc Tukey’s HSD (Honestly Significant Difference) test

Several target genes were pinpointed in this study to elucidate photoperiod-mediated GA biosynthetic and signaling response pathways related to sex differentiation. Among these, GRAS (*GAI*, *RGA*, *SCR*) family members were significantly enriched in PPS_LD. SCLs and DELLA proteins belonged to a subfamily of the plant-specific GRAS family [44, 45]. Moreover, SCLs antagonized with DELLA in controlling both downstream GA responses and upstream GA biosynthetic genes [46]. GAI (GA insensitive) and RGA (repressor of ga1-3) served as inhibitors or negative regulators of the GA signaling [47]. SCARECROW-LIKE 3 was indicated as a positive regulator of GA signaling [46], and SCL13 and SCL21 were vital in the signal transduction of phytochrome A [48]. In this study, the expression levels of *CmSCL3*, *CmSCL13*, *CmSCL14*, *CmSCL15*, *CmSCL4-like*, and *CmSCL34* genes were higher in PPS_LD, which was associated with an absence of female flowers compared with the other three groups. On the contrary, DELLA proteins (*CmGAI*: *CmoCh11G005830*) showed lower expression in PPS_LD compared with the other three groups (Fig. 5, Table S5). Regarding the GA biosynthetic pathway, few genes were observed with differential expression between PPS_LD and the other groups. In addition, photoperiod-spanning LDs were found not to induce an increase in the GA content in either PPS or PPIS pumpkin lines (Fig. 6). Furthermore, GA-regulated protein 9-like, *CmGASA9* (*CmGRP9*), which is a member of the GASA family involved in regulating floral meristem and floral organ identity, was significantly and highly expressed in PPS_LD compared with the other three groups [49, 50]. This suggested that the GA signaling pathway might regulate the photoperiodic flowering network rather than the synthetic pathway.

### DEGs associated with the ethylene biosynthetic and ethylene response pathways

Most of the sex-determining genes encompass ethylene biosynthesis (ACS and ACO multigene enzymes), ethylene perception (ethylene receptors, or ETRs) and ethylene response factors (ERFs). The expression of numerous ERFs was found to be significantly lower in PPS_LD compared with the other three groups, consistent with the reduced female flowering observed in PPS_LD, as ERFs act as positive regulators in the ethylene signaling pathway. Some examples of these ERFs include *CmERF118*, *CmERF118-like1,2*, *CmWIN1-like*, and *CmRAP2-7-like*. In addition, ethylene biosynthetic pathway–associated gene ACC oxidase (*CmACO3* and *CmACO1*) exhibited a noticeable downregulation in PPS_LD compared with the other three groups. This suggested that long photoperiods may mediate ethylene synthesis and ethylene response, thereby affecting the differentiation of female flowers (Fig. 5, Table S5).

### Validation of gene expression patterns by real-time quantitative PCR

**Fig. 7:**
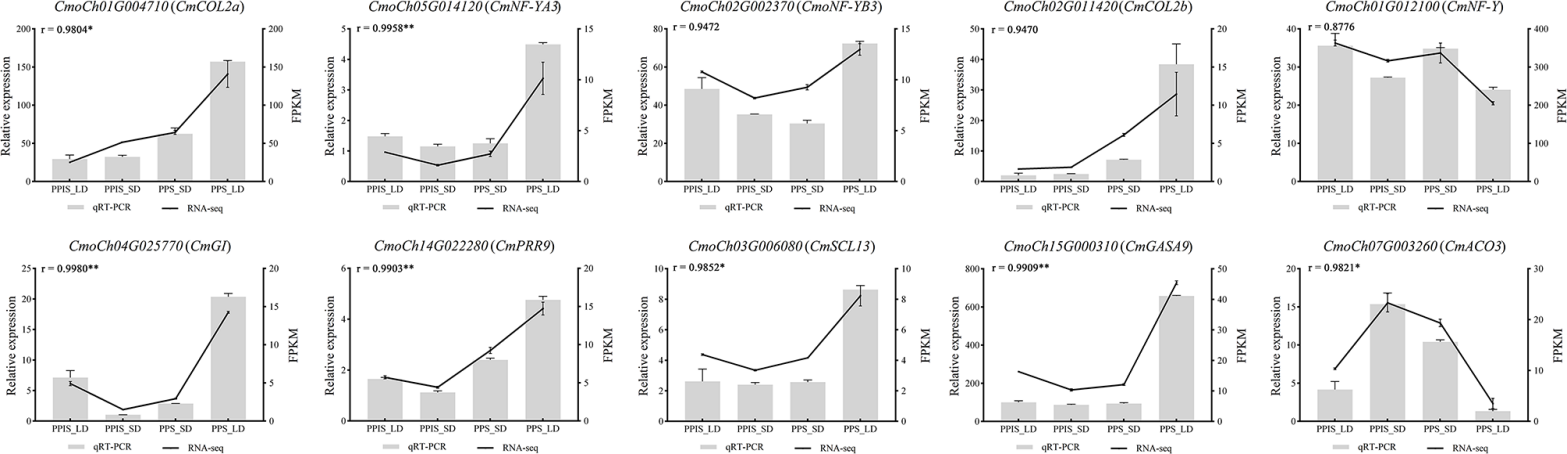
Validation of gene expression patterns by real-time quantitative PCR

Ten genes related to sex differentiation were selected in this study for qRT-PCR analysis to validate the RNA-seq findings. The details of the genes and primer pairs used in this study are presented in Table S1. The comparative analysis of qRT-PCR and RNA-seq data for these genes revealed a high degree of consistency in their expression patterns, with an average r-value of 0.9 (the r-value varied from 0.88 to 1.00), affirming the accuracy and reliability of the transcriptome analysis (Fig. 7).

## Discussion

Photoperiod, which refers to the length of the day, is a significant environmental signal that has been extensively investigated for its role in regulating flowering in many plants, such as soybeans and rice [19, 51, 52]. However, in these crops, photoperiod mainly regulates the timing of flowering (the differentiation of the floral buds) and is not directly involved in sex differentiation. In the Cucurbitaceae family, the floral meristem undergoes the successive initiation of sepal, petal, stamen, and carpel primordia at the early bud stage. Subsequently, it develops into a female or male flower by the arrest of either stamen or carpel development [7]. The Cucurbitaceae family comprises hermaphrodite species, where sex inheritance is essential in cucurbit breeding. Genetic and environmental factors mainly influence this process [11]. Therefore, investigating how photoperiod regulates sex differentiation in cucurbits is of great significance.

*Cucurbita* encompasses three extensively cultivated species, *Cucurbita pepo*, *C. moschata*, and *C. maxima*, exhibiting a broad spectrum of phenotypic diversity [53]. In China, *C. moschata* Duch is the most prominent among the three cultivated species. However, *C. moschata* is an SD plant with unisexual flowers characterized by photoperiod-sensitive traits. Within this species, the “miben” pumpkin, as the main type of *C. moschata*, possesses several advantages, including stress resistance, high yield, and significant lutein content. Therefore, overcoming its sensitivity to photoperiod is essential for promoting extensive cultivation and reducing transportation costs. Previous studies conducted high-density QTL mapping for two key traits: early flowering and photoperiod insensitivity in *C. moschata*, both of which are of paramount importance to plant breeders [27, 28]. Regarding the photoperiod-sensitive trait, a QTL to chromosome 10 was successfully mapped, encompassing 73 genes identified through SLAF-seq technology [27]. In the case of early flowering, two key genes were identified, namely “flowering locus T-like protein” and “flowering locus T-like 2,” which are associated with the first female and first male flowering node, located on chromosomes 10 and 11, respectively [28]. Some linked inDel markers were used for Marker-Assisted Breeding (MAS) [27, 28]. Regarding *Cucurbita pepo*, numerous genes associated with sex determination have been reported, mostly related to ethylene synthesis and perception. These include genes such as *CpACS27A*, *CpACO1A*, *CpETR1A*, and *CpETR2B* [54–58]. However, there have been limited studies on photoperiod-mediated sex differentiation in pumpkins.

This study carried out a phenotypic analysis of PPS and PPIS pumpkin lines exposed to different day lengths (LD and SD). The findings of this study revealed that female flower differentiation was markedly inhibited in PPS_LD. However, no significant differences were observed in the other three groups (PPS_SD, PPIS_LD, and PPIS_SD). Transcriptome analysis for these four groups was performed to gain deeper insights into the potential dominant genes involved in the photosensitive regulation of sex differentiation. First, based on PCA, it became evident that PPS_SD, PPIS_LD, and PPIS_SD formed a cluster distinct from PPS_LD. Second, hierarchical cluster analysis revealed that DEGs could be classified into six gene subclusters, with gene subclusters 1, 3, and 5 showing differential expression in PPS_LD. When coupled with functional annotations and enrichment analysis, significant upregulation of photoreceptors (*CmCRY1*, *F-box/kelch-repeat protein*), circadian rhythm–related genes (*CmGI*, *CmPRR9*, *CmRUP2*, and *adagio protein 3*), and CONSTANS (*CmCOL2a*, *CmCOL2b*, and *CmCOL5*) in PPS_LD was observed. Meanwhile, the majority of NF-Y TFs exhibited a pronounced downregulation.

Previous studies indicated that the photoreceptor FLAVIN-BINDING, KELCH-REPEAT, F-BOX 1 (FKF1) protein can stabilize and interact with the CONSTANS (CO) protein, controlling flowering timing in LD plants like *Arabidopsis* [20, 59]. In contrast, the circadian rhythm-related gene PRR-like protein (OsPRR37) delays flowering under LD conditions in SD plants like rice [60]. LD and SD plants employ different mechanisms in response to photoperiod. In this study on *C. moschata* (a SD plant), *CmPRR9* with upregulation in PPS_LD displayed a delayed female flower initiation phenotype consistent with rice.

NF-Y are positive regulators of photomorphogenesis in *Arabidopsis thaliana* involved in both light and GA signaling to promote flowering [39, 40, 61, 62]. NF-Y interacts with CONSTANS in the photoperiod pathway and with DELLAs in the GA pathway, directly influencing the transcription of SOC1, a significant integrator of floral pathways [62]. Furthermore, in Xishuangbanna (XIS) cucumbers, which are strict SD cucurbits, *NF-YA1* is the major QTL effect site regulating sex differentiation [29]. This study revealed significant distinctions in the expression of NF-Y family genes in the PPS_LD group compared with the other groups, suggesting the possibility of photoperiod-mediated regulation of sex differentiation by NF-Y TFs.

CONSTANS (CO) serves as a crucial regulator that controls the levels of *FLOWERING LOCUS T* transcripts. It plays a central role in the photoperiodic flowering pathway by integrating various external and internal signals [21]. In *Arabidopsis* (an LD plant), CO induces the expression of *FT* under LDs, thus promoting flowering. In contrast, in rice (an SD plant), CO homologs have been suggested to have opposing roles in flowering time regulation: they promote flowering under inductive SD conditions while delaying it under LD conditions [43, 63, 64]. In this study on *C. moschata*, CONSTANS (*CmCOL2a*, *CmCOL2b*, and *CmCOL5*) exhibited significant upregulation in PPS_LD, which displayed delayed female flower differentiation compared with the other three groups. In addition, a decrease in the expression of *FT* (*CmFTL2*) in PPS_LD was discovered, which significantly suppressed female flower determination. This finding aligned with previous research conducted on rice [64].

The photoperiod can influence the GA pathway, thereby impacting sex differentiation in plants. Molecular genetic analyses of the GA and photoperiod pathways have indicated a synergistic promotion of flowering under LD conditions [25, 65]. However, previous research findings suggested that the photoperiod did not influence the GA content, and the expression of pivotal genes involved in GA metabolism, specifically GA20 oxidase (*GA20ox*) and GA2 oxidase (*GA2ox*), showed no significant differences [66]. However, members of the GRAS family, SCL and DELLA proteins, which were involved in signal transduction pathways, were significantly enriched. GAI (GA insensitive) and RGA (repressor of ga1-3) served as inhibitors or negative regulators of the GA signaling [47, 67, 68]. SCL3 was considered a positive regulator of GA signaling [46]. This study observed upregulation of positive GA regulatory factors, including *CmSCL3*, *CmSCL13*, *CmSCL14*, *CmSCL15*, *CmSCL4-like*, and *CmSCL34*, in PPS_LD compared with the other three groups. Conversely, the DELLA protein (*CmGAI: CmoCh11G005830*), a negative regulator of GA signaling, exhibited downregulation in PPS_LD compared with the other groups, and this was associated with a phenotype characterized by inhibited female flower differentiation. Therefore, it was inferred that under long photoperiod conditions, the GA signaling pathway was positively activated, promoting the differentiation of flower buds into male flowers and inhibiting the production of female flowers.

Furthermore, it has been documented that shorter day lengths stimulate ethylene production by accelerating the expression of genes involved in ethylene synthesis, resulting in an increased occurrence of female flowers in cucumbers [3]. Ethylene plays an essential role in regulating sexual differentiation in cucurbits. It halts stamen development and fosters carpel development, controlling the transition from male to female phases. Several key genes related to ethylene synthesis, receptors, and signal response, such as *CsACS2*, *CsACO3*, *CsETR1*, *CsCaN*, *CsPIF4*, and *AP2/ERF*, actively govern female flower differentiation in response to photoperiod modulation [6, 17, 69, 70]. In this study, under LD conditions, no noteworthy difference was observed in the expression of ethylene receptor genes in the photoperiod-sensitive germplasm. However, significant differences were observed in the expression of ethylene synthesis genes, such as *ACOs*, and signal response genes, specifically *ERFs*, including *CmERF118*, *CmERF118-like1*,*2*, *CmWIN1-like*, and *CmRAP2-7-like*.

## Conclusions

**Fig. 8:**
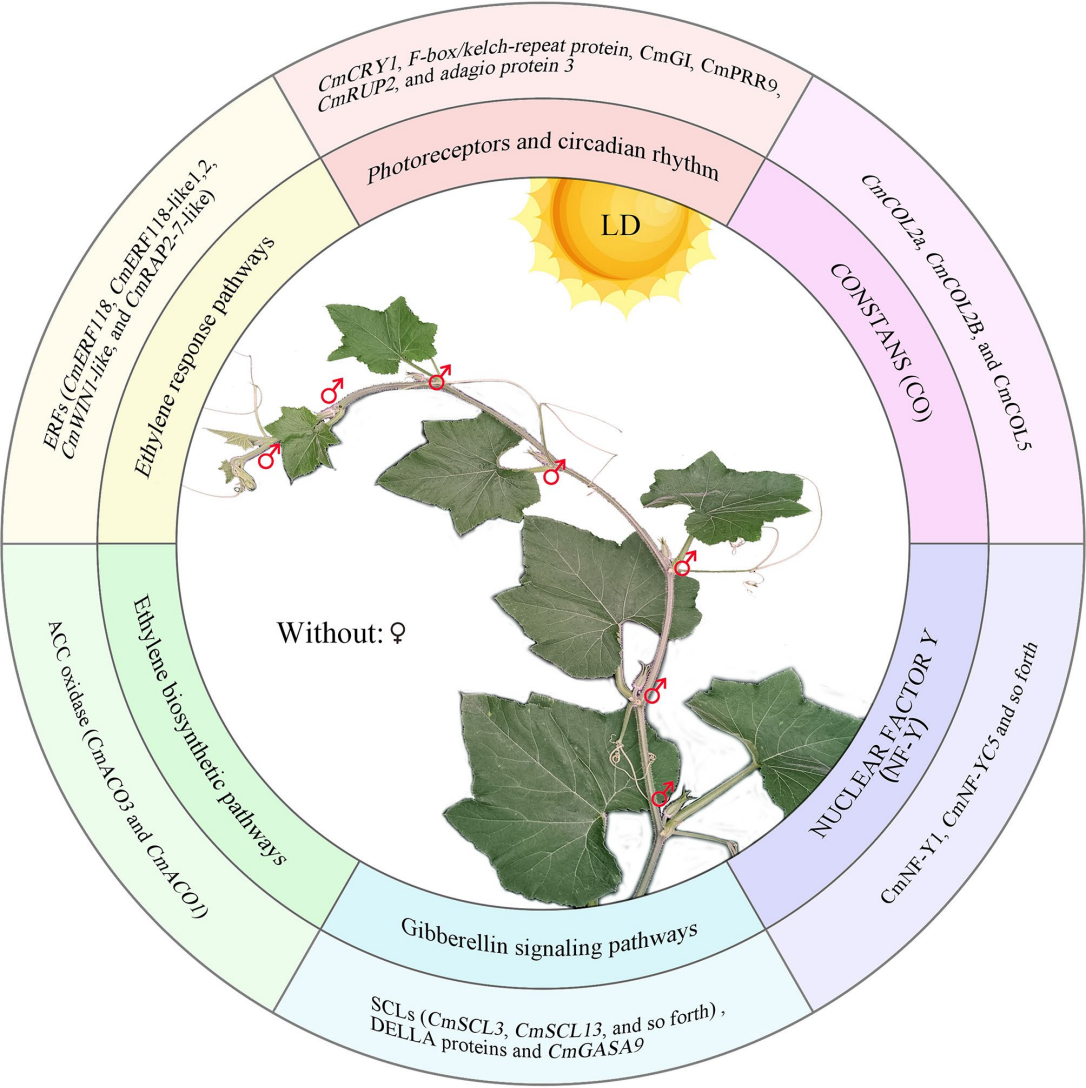
Schematic diagram of the molecular basis of photoperiod-regulated sex differentiation in pumpkin

The photoperiod insensitivity of pumpkin (*C. moschata* Duch.) associated with sex differentiation may be attributed to the coordinated regulation of multiple flowering pathways and signaling molecules. These include the photoperiod-mediated flowering pathway involving genes such as *CmPRR9*, CONSTANS (*CmCOL2a*, *CmCOL2b*, and *CmCOL5*), and NF-Y TFs. Additionally, the GA signaling pathway was involved, with key members such as GRAS family proteins SCL and DELLA. Moreover, ethylene synthesis genes (*ACOs*) and signal response genes (*ERFs*) were crucial in this process (Fig. 8). This study provided insights for a deeper understanding of the photoperiod regulatory mechanisms in miben pumpkin, offering a basis for further exploration of gene loci involved in regulating photoperiod insensitivity.

### Electronic supplementary material

Below is the link to the electronic supplementary material.

Supplementary Material 1

Supplementary Material 2

## Data Availability

All the data used in this manuscript are available online and can be checked on Nation Centre for Biotechnology Information (NCBI), PRJNA1041978.
