# Supplementary material for: Transcriptomic analysis reveals the molecular basis of photoperiod-regulated sex differentiation in tropical pumpkins (Cucurbita moschata Duch.)
Source: BMC Plant Biol. 2024 Feb 6;24:90. doi: 10.1186/s12870-024-04777-3 (PMC10845594; doi:10.1186/s12870-024-04777-3)
Supplement: Supplementary file 1 — Supplementary Material 1 [file 12870_2024_4777_MOESM1_ESM.docx]

**Additional file 1: Supplementary Table 1.** Primers used in qRT-PCR. **Supplementary Table 2.** Summary of sequencing data generated and mapping to the C. moschata transcriptome. **Supplementary Table 3.** All DEGs identified among four samples (PPS_LD, PPS_LD, PPIS_SD, and PPIS_LD). **Supplementary Table 4.** DEGs identified among subclusters 1, 3, and 5 from hierarchical clustering analysis. **Supplementary Table 5.** DEGs related to photoperiod-regulated sex differentiation.
